# Extracellular Reactive Oxygen Species (ROS) Production in Fresh Donkey Sperm Exposed to Reductive Stress, Oxidative Stress and NETosis

**DOI:** 10.3390/antiox10091367

**Published:** 2021-08-27

**Authors:** Iván Yánez-Ortiz, Jaime Catalán, Yentel Mateo-Otero, Marta Dordas-Perpinyà, Sabrina Gacem, Natalia Yeste, Anna Bassols, Marc Yeste, Jordi Miró

**Affiliations:** 1 Equine Reproduction Service, Department of Animal Medicine and Surgery, Faculty of Veterinary Sciences, Autonomous University of Barcelona, E-08193 Bellaterra, Cerdanyola del Vallès, Spain; ivan.yanez22@gmail.com (I.Y.-O.); dr.jcatalan@gmail.com (J.C.); martadordas@gmail.com (M.D.-P.); swp.sabrina.gacem@gmail.com (S.G.); 2 Biotechnology of Animal and Human Reproduction (TechnoSperm), Institute of Food and Agricultural Technology, University of Girona, E-17003 Girona, Spain; yentel.mateo@udg.edu; 3 Unit of Cell Biology, Department of Biology, Faculty of Sciences, University of Girona, E-17003 Girona, Spain; 4 Department of Biochemistry and Molecular Biology, Faculty of Veterinary Sciences, Autonomous University of Barcelona, E-08193 Bellaterra, Cerdanyola del Vallès, Spain; nati31194@gmail.com (N.Y.); anna.bassols@uab.cat (A.B.)

**Keywords:** reactive oxygen species (ROS), reduced glutathione (GSH), hydrogen peroxide (H_2_O_2_), polymorphonuclear neutrophils (PMN), NETosis, seminal plasma (SP), donkey

## Abstract

Jenny shows a large endometrial reaction after semen influx to the uterus with a large amount of polymorphonuclear neutrophils (PMN) migrating into the uterine lumen. PMN act as a sperm selection mechanism through phagocytosis and NETosis (DNA extrudes and, together with proteins, trap spermatozoa). While a reduced percentage of spermatozoa are phagocytosed by PMN, most are found to be attached to neutrophil extracellular traps (NETs). This selection process together with sperm metabolism produces a large amount of reactive oxygen species (ROS) that influence the reproductive success. The present study aimed to determine the extracellular ROS production in both sperm and PMN. With this purpose, (1) donkey sperm were exposed to reductive and oxidative stresses, through adding different concentrations of reduced glutathione (GSH) and hydrogen peroxide (H_2_O_2_), respectively; and (2) PMN were subjected to NETosis in the presence of the whole semen, sperm, seminal plasma (SP) or other activators such as formyl-methionyl-leucyl-phenylalanine (FMLP). Extracellular ROS production (measured as H_2_O_2_ levels) was determined with the Amplex^®^ Red Hydrogen Peroxide/Peroxidase Assay Kit. Donkey sperm showed more resilience to oxidative stress than to the reductive one, and GSH treatments led to greater H_2_O_2_ extracellular production. Moreover, not only did SP appear to be the main inducer of NETosis in PMN, but it was also able to maintain the extracellular H_2_O_2_ levels produced by sperm and NETosis.

## 1. Introduction

Donkey reproductive strategy shows a large spermatogenic efficiency in the male [1] with very high sperm concentration, motility and viability values [2], and a high inflammatory response in the endometrium after Artificial Insemination (AI) in the female [3]. In fact, it has been established that sperm are the main factors responsible for the endometrial physiological inflammation that occurs in the donkey after mating (natural breeding or AI) [4], which has also been observed in other species [5,6,7,8]. This triggers the activation of the defense cells of the immune system, specifically polymorphonuclear neutrophils (PMN), which move rapidly toward the endometrium to counteract the inflammatory process generated by the presence of sperm, which is necessary for pregnancy to be established [9]. This influx of PMN is particularly higher and even faster in jennies than in mares, reaching its maximum concentration between 6–12 h post-AI, which may underlie a lower probability of fertilization [4,10].

According to Branzk et al. [11], as a result of infection, the action of PMN can occur through two ways depending on the size of the pathogen. Indeed, not only can PMN eliminate infectious agents by phagocytosis, but they also have the ability to degranulate/extrude their DNA and bactericidal molecules (histones and enzymes) and form neutrophil extracellular traps (NETs), which are found in the extracellular space and create a unique type of cell death called NETosis [12,13]. Kotilainen et al. [14] reported that infusing spermatozoa rather than bacteria into the mare uterus provokes the influx of PMN. Within this context, Miró et al. [10] recently observed in donkeys that sperm:PMN binding could inhibit the first action of PMN, i.e., phagocytosis, which would lead to the activation of the second action of PMN, i.e., NETosis.

In the horse, it has been previously established that although the production of reactive oxygen species (ROS) is a consequence of the sperm metabolism itself [15,16], the generation of energy in the form of mitochondrial adenosine triphosphate (ATP) depending on oxidative phosphorylation (OXPHOS) [17,18], the action of PMN is also an important source of ROS generation in the extracellular medium [19,20]. In the horse, Baumber et al. [21] observed a significant increase (300–400 times more) in the production of hydrogen peroxide (H_2_O_2_), one of ROS molecules, by sperm incubated with PMN, compared with the amount of H_2_O_2_ produced by sperm only. This large amount of H_2_O_2_ produced by PMN causes a significant decrease (35–40%) in sperm motility [21]. However, these values in the donkey are still unknown.

Physiologically, the enzymatic and non-enzymatic antioxidants found in the seminal plasma (SP) are responsible for controlling the ROS produced by sperm [22], thus establishing a physiological homeostasis in the redox balance between ROS and antioxidant systems in sperm [23]. The imbalance can give rise to two situations, both with detrimental effects on sperm motility and viability [24]. The first one is a reductive stress state, due to lower ROS production or the change in the redox balance in favor of antioxidants. The second is an oxidative stress state, caused by an excessive ROS production or a decrease in the activity of antioxidants [25].

Papas et al. [26] demonstrated that the capacity of the enzymatic antioxidants present in the SP is higher in the donkey than in the horse. It is known that jenny PMN exposed to donkey sperm undergo NETosis [27], and that NETosis produces ROS [28]. On the other hand, SP modulates sperm-PMN binding [29] and is able to induce NETosis in the donkey [30]. Our hypothesis is that the amount of extracellular ROS (measured as H_2_O_2_ levels) produced by PMN during NETosis in the presence of sperm/SP is significantly higher than that produced in the presence of sperm without SP and that, for this reason, donkey sperm have higher capacity to withstand oxidative stress. Therefore, the objectives of this study were (1) to determine the amount of extracellular ROS produced by donkey sperm subjected to reductive and oxidative stresses, and (2) to assess the amount of extracellular H_2_O_2_ produced by NETosis.

## 2. Materials and Methods

### 2.1. Animals and Statement of Ethics

The study was performed with sperm samples obtained from four separate Catalonian jackasses between 3–6 years old, and with blood samples obtained from three Catalonian jennies aged 3–8 years old. All animals were clinically healthy, with a good body condition and of proven fertility (males with good fertility rates and females with at least one foaling). All animals were housed at the Equine Reproduction Service, Autonomous University of Barcelona (Bellaterra, Cerdanyola del Vallès, Spain). This is an equine sperm collection center approved by the European Union (EU), with authorization number ES09RS01E. All animal handling protocols in the center include strict animal health and welfare controls. Jackasses were maintained in individual paddocks and jennies were grouped in a big paddock (maximum 10 animals per paddock). Their diet was based on grain, straw and hay fodder, in addition to water ad libitum. The sperm collection was carried out under the sanitary guidelines established by the Council of the European Communities in Directive 82/894/CEE of 21 December 1982, which includes that housed animals are free of equine viral arteritis, equine infectious anemia and equine contagious metritis. The study was approved by the Ethics Committee of the Autonomous University of Barcelona (authorization code: CEEAH 1424).

### 2.2. Experimental Design

Two separate experiments were performed: (1) sperm exposure to reductive and oxidative stresses, and (2) determination of extracellular H_2_O_2_ production by NETosis.

### 2.3. Experiment 1. Sperm Exposure to Reductive Stress and Oxidative Stress

#### 2.3.1. Sperm Collection

The jackasses were collected on a regular schedule in the morning. Sperm were collected through an artificial vagina (Hannover model; Minitüb GmbH, Tiefenbach, Germany) preheated to a temperature between 48 °C–50 °C and coupled with an in-line nylon filter to remove the gel fraction. Immediately after collection, each ejaculate was split into two fractions: one was intended to recovering the SP and the other was diluted 1:5 (*v*:*v*) in a cooling extender based on skimmed milk [31], previously tempered to 37 °C. Sperm concentration of diluted semen was evaluated by a Neubauer chamber (Paul Marienfeld GmbH & Co. KG, Lauda-Königshofen, Germany) and adjusted to 100 × 10^6^ sperm/mL.

#### 2.3.2. Seminal Plasma (SP) Collection

The SP fraction of each ejaculate was obtained by successive centrifugations at 3000× *g* and 4 °C for 10 min (Medifriger BL-S; JP Selecta S.A., Barcelona, Spain). The process was repeated under the same conditions as many times as necessary (~5 depending on the ejaculate) until sperm were completely eliminated. Verification was carried out using a phase contrast microscope (Olympus Europa SE & Co. KG, Hamburg, Germany) at 200× magnification. For all experiments, SP samples were maintained in a water bath at 37 °C until used.

#### 2.3.3. Treatments

Ten treatments were prepared with the sperm sample obtained from each jackass and with the SP obtained from each jackass in a final volume of 1.5 mL as follows: T1 = Sperm and SP (control); T2 = Sperm and SP + 2 mM (final concentration) reduced glutathione (GSH; G4251; Sigma-Aldrich, St. Louis, MO, USA); T3 = Sperm and SP + 4 mM GSH; T4 = Sperm and SP + 6 mM GSH; T5 = Sperm and SP + 8 mM GSH; T6 = Sperm and SP + 10 mM GSH; T7 = Sperm and SP + 0.5 mM hydrogen peroxide (H_2_O_2_; 30% (*w*:*w*); 7722-84-1; Sigma-Aldrich, St. Louis, MO, USA); T8 = Sperm and SP + 1 mM H_2_O_2_; T9 = Sperm and SP + 5 mM H_2_O_2_; and T10 = Sperm and SP + 10 mM H_2_O_2_.

All treatments were incubated in a water bath at 37 °C and analyzed after 0 min, 30 min, 60 min and 120 min of incubation. All experiments were carried out by the same technician to avoid errors and biases due to the human factor. Sperm motility evaluation was performed using a computer-assisted sperm analysis (CASA) system (Proiser R + D, Valencia, Spain), and sperm viability was assessed through eosin-nigrosin staining. Extracellular ROS production (measured as H_2_O_2_ levels) was determined by the Amplex^®^ Red Hydrogen Peroxide/Peroxidase Assay Kit (A22188; ThermoFisher Scientific, Waltham, MA, USA).

#### 2.3.4. Evaluation of Sperm Motility

Objective evaluation of sperm motility was performed using the motility module of the CASA ISAS^®^ V 1.2 system (CASA-Mot; Proiser R + D, Valencia, Spain) combined with a 10× negative phase contrast microscope (UOP200i; Proiser R + D, Valencia, Spain). A high-resolution digital camera (MQ003MG-CM; Proiser R + D, Valencia, Spain), capable of capturing up to 100 frames per second (fps), was used. Briefly, 2 µL of each sperm sample was placed into a reusable counting chamber of 10 µm in depth (Spermtrack^®^10; Proiser R + D, Valencia, Spain), previously heated to 37 °C. A minimum of 500 spermatozoa was counted per analysis. In each evaluation, total (TM, %) and progressive motility (PM, %) were recorded, as well as the following kinematic parameters: curvilinear velocity (VCL, µm/s), straight line velocity (VSL, µm/s), average path velocity (VAP, µm/s), linearity coefficient (LIN = [VSL/VCL] × 100, %), straightness coefficient (STR = [VSL/VAP] × 100, %), wobble coefficient (WOB = [VAP/VCL] × 100, %), amplitude of lateral head displacement (ALH, µm) and beat-cross frequency (BCF, Hz). The CASA-Mot settings were those recommended by the manufacturer: particle area > 4 and <75 µm^2^, connectivity = 6, minimum images number to calculate ALH = 10. The cut-off values for total and progressively motile sperm were VAP ≥ 10 µm/s and STR ≥ 75%, respectively. Three technical replicates were evaluated.

#### 2.3.5. Evaluation of Sperm Viability

Sperm viability was determined through eosin-nigrosin staining [32]. Briefly, 10 µL of each sperm sample was placed on a slide, previously warmed to 37 °C, and 10 µL of the eosin-nigrosin stain was added. Subsequently, a smear of the mixture was prepared on the slide using a glass rod. Samples were air dried at room temperature. A minimum of 200 sperm/sample were evaluated under a bright field optical microscope (Carl Zeiss, Göttingen, Germany) at 1000× magnification using immersion oil. Three technical replicates were evaluated, and the percentage of viable spermatozoa (eosin negative) was recorded.

#### 2.3.6. Determination of Extracellular H_2_O_2_ Production

The generation of extracellular H_2_O_2_ was determined through the Amplex^®^ Red Hydrogen Peroxide/Peroxidase Assay Kit (A22188; ThermoFisher Scientific, Waltham, MA, USA), a highly sensitive and stable probe for measuring H_2_O_2_ production.

The detection system consisted of the dye, 10-acetyl-3,7-dihydroxyphenoxazine (Amplex^®^ Red), horseradish peroxidase (HRP) and 50 mM sodium phosphate (pH = 7.4). In this system, 100 µM Amplex Red reagent in the presence of 0.2 U/mL HRP reacts with H_2_O_2_ in a 1:1 (*v*:*v*) stoichiometry to produce a highly red-fluorescent oxidation product, resorufin, that can be measured by fluorescence or absorbance.

The Amplex^®^ Red assay was performed on each sample according to the manufacturer’s instructions. Treatments and assay standards (final H_2_O_2_ concentrations: 0 µM, 0.5 µM, 1 µM, 2 µM, 2.5 µM, 4 µM, 5 µM, 8 µM, 10 µM and 20 µM) were pipetted into a 96-well microplate with duplicates for each sample. Absorbance was measured using spectrophotometry at 560 nm and monitored at multiple time points, every 30 min for 2 h, to follow the kinetics of the reaction at 37 °C. Results are expressed as a single blank-corrected concentration.

### 2.4. Experiment 2. Determination of Extracellular H_2_O_2_ Production by NETosis

#### 2.4.1. Samples

Sperm were obtained as explained in Section 2.3.1. For this experiment, sperm concentration was adjusted to 1.5 × 10^6^ sperm/mL. SP was harvested as described in Section 2.3.2.

#### 2.4.2. PMN Isolation

For isolating PMN from the peripheral blood, the protocol set by Siemsen et al. [33] and Yildiz et al. [34] was followed. Briefly, five blood samples were collected through jugular venipuncture in 10 mL BD Vacutainer^®^ tubes containing anticoagulant (18.0 mg of ethylenediaminetetraacetic acid; EDTA; BD, Plymouth, UK). Immediately afterward, blood samples were incubated in a water bath at 37 °C for 30 min to separate red blood cells (RBC) and the plasma fraction rich in leukocytes. The fraction of 5 mL above the red blood cell (RBC) pellet was recovered and mixed with an equal volume of 0.02% EDTA solution (E8008; Sigma-Aldrich, St. Louis, MO, USA). In turn, this mixture was layered in sterile tubes with an equal volume of Ficoll^®^ Paque Plus (GE17-1440-02; Merck KGaA, Darmstadt, Germany) and centrifuged at 500× *g* and 20 °C for 30 min (Medifriger BL-S; JP Selecta S.A., Barcelona, Spain) to extract blood plasma, lymphocytes and monocytes. The pellet containing RBC and PMN was mixed with 25 mL of sterile PBS 1× and centrifuged at 500× *g* and 20 °C for 10 min. The fresh pellet obtained from this second centrifugation was resuspended in RBC 1× lysis buffer (00-4333-57; ThermoFisher Scientific, Waltham, MA, USA) following the manufacturer’s instructions. Subsequently, samples were gently shaken for 15 min, and 25 mL of sterile PBS 1× was added. The resulting mixture was centrifuged again (500× *g* and 20 °C for 10 min), and the pellet was resuspended in 1 mL RPMI-1640 medium (R0883; Sigma-Aldrich, St. Louis, MO, USA) supplemented with Penicillin-Streptomycin at 1% (P4333; Sigma-Aldrich, St. Louis, MO, USA). Finally, samples were analyzed using a hematological flow cytometer (Sysmex XN-1000™; Sysmex Corporation, Kobe, Japan) to evaluate the purity and efficiency of the isolation method, and only those having >80% PMN were used to test the treatments. Concentration of PMN was adjusted to 3 × 10^5^ PMN/mL.

#### 2.4.3. Treatments

Six treatments were prepared in a final volume of 150 µL as follows: T1 = PMN (control): 75 µL of PMN + 75 µL of RPMI-1640 medium [30]; T2 = PMN + Whole semen (sperm + SP): 75 µL of PMN + 75 µL of Whole semen [10]; T3 = PMN + Sperm: 75 µL of PMN + 75 µL of Sperm [30]; T4 = PMN + SP: 75 µL of PMN + 37.5 µL of SP (25% of SP with respect to the total volume) + 37.5 µL of RPMI-1640 medium [30]; T5 = PMN + formyl-methionyl-leucyl-phenylalanine (FMLP): 75 µL of PMN + 15 µL 0.1 mM FMLP (F3506; Sigma-Aldrich, St. Louis, MO, USA) + 60 µL of RPMI-1640 medium [10,21,30]; and T6 = PMN + Kenney: 75 µL of PMN + 75 µL of Kenney [30].

All treatments were incubated at 37 °C for 2 h. After that, PMN were stained with SYTOX™ Orange Nucleic Acid Stain (S11368; ThermoFisher Scientific, Waltham, MA, USA) and the percentage of reacted PMN (NETosis) was evaluated using a confocal microscope. Extracellular ROS production (measured as H_2_O_2_ levels) was determined through the Amplex^®^ Red Hydrogen Peroxide/Peroxidase Assay Kit (A22188; ThermoFisher Scientific, Waltham, MA, USA). The evaluations were carried out by the same technician to avoid errors and biases due to the human factor.

#### 2.4.4. Evaluation of NETosis

NETosis was evaluated following the protocol established by Mateo-Otero et al. [30]. In brief, treatments were incubated at 37 °C for 2 h in sterile Millicell^®^ EZ SLIDES 8-well plates (Merck KGaA, Darmstadt, Germany). Thereafter, 200 µL of 4% Paraformaldehyde (158127; Sigma-Aldrich, St. Louis, MO, USA) was added to each well to fix the cells, and three washes were performed with 200 µL of sterile PBS 1×. Subsequently, 150 µL of SYTOX™ Orange Nucleic Acid Stain (S11368; ThermoFisher Scientific, Waltham, MA, USA) diluted 1:2500 (*v*:*v*) was placed in each well to stain the cells for 30 min in darkness. Finally, cells were washed with sterile PBS 1× before mounting them on slides with DPX mounting medium (06522; Sigma-Aldrich, St. Louis, MO, USA). A minimum of 200 cells/treatment were evaluated with a confocal laser scanning microscope (Olympus FluoView™ FV1000; Olympus Corporation, Tokyo, Japan) at 200× magnification. The percentage of reacted PMN (exhibiting expanded or elongated nuclei) was recorded, and two technical replicates were counted.

#### 2.4.5. Determination of Extracellular H_2_O_2_ Production

Evaluation of extracellular H_2_O_2_ production was carried out as explained in Section 2.3.6.

### 2.5. Statistical Analysis

Data obtained from all experiments are included in the section of Supplementary Materials and were analyzed with the statistical package R (V 4.0.3, R Core Team; Vienna, Austria) and plotted using GraphPad Prism (V 8.4.0, GraphPad Software LLC; San Diego, CA, USA). At first, normality of data was verified through the Shapiro–Wilk test and homoscedasticity using the Levene test. When necessary, data were linearly transformed with arcsin √x. When, even transformed, data did not present normal distribution and/or variances were not homogenous, a non-parametric analysis was performed using the Friedman test followed by the Wilcoxon test for pairwise comparison.

In experiment 1, sperm motility and viability were compared with a generalized linear model mixed (GLMM; repeated measures). The within-subjects factor was the incubation time (0 min, 30 min, 60 min and 120 min), the fixed-effects factor was the concentration of GSH (control, 2 mM, 4 mM, 6 mM, 8 mM and 10 mM) or H_2_O_2_ (control, 0.5 mM, 1 mM, 5 mM and 10 mM) and the random-effects factor was the jackass. The extracellular H_2_O_2_ production by sperm and SP in the different GSH and H_2_O_2_ concentrations was compared by a two-way ANOVA. In experiment 2, the percentage of reacted PMN in the different treatments (PMN [control], PMN + Whole semen, PMN + Sperm, PMN + SP, PMN + FMLP and PMN + Kenney) was compared by one-way ANOVA. The extracellular H_2_O_2_ production by NETosis in the different treatments was analyzed with a GLMM, including the time of incubation (0 min, 30 min, 60 min, 90 min and 120 min) as a within-subject factor and considering the jenny as a random factor. The Bonferroni post hoc test was used for pair-wise comparisons. The differences were considered to be statistically significant when *p* ≤ 0.05. Results are expressed as means ± standard error of the mean (SEM).

## 3. Results

### 3.1. Experiment 1: Exposure of Sperm to Reductive Stress

#### 3.1.1. Sperm Motility

**Figure 1:**
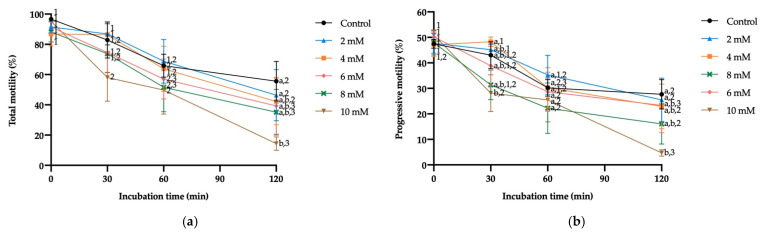
Mean ± SEM of total (**a**) and progressive (**b**) motility of donkey sperm exposed to reductive stress with different reduced glutathione (GSH) concentrations measured at distinct incubation times. (a,b) Different letters indicate significant differences (*p* ≤ 0.05) between GSH concentrations within a given incubation time. (1–3) Different numbers indicate significant differences (*p* ≤ 0.05) between incubation times within a given GSH concentration.

No significant differences in TM and PM were observed when sperm were exposed to a GSH concentration of 8 mM, at any of the incubation times. However, 10 mM GSH was seen to reduce TM and PM after 120 min of incubation (*p* ≤ 0.05). On the other hand, the impact of GSH on TM and PM was concentration-dependent; the higher the concentration, the earlier the reduction in these two motility parameters (30 min for 10 mM GSH; 60 min for 4 mM, 6 mM and 8 mM GSH; 120 min for the control and 2 mM GSH; Figure 1a,b).

Regarding kinematic parameters (Table 1), the different GSH concentrations had no significant effect on VCL, VSL, VAP, STR or ALH at any of the incubation times. However, the treatment containing 8 mM GSH showed significantly lower LIN after 30 and 120 min of incubation (*p* ≤ 0.05). Similarly, WOB was significantly lower in the treatments containing 6 mM and 8 mM GSH after 30 min and 120 min of incubation, respectively. Finally, the presence of 8 mM reduced BCF after 30 min, 60 min and 120 min of incubation (*p* ≤ 0.05).

As far as the effects of incubation time on motility are concerned, kinematic parameters did not vary significantly in treatments containing 2 mM and 6 mM GSH, except for WOB and BCF (*p* ≤ 0.05). At the other concentrations, the incubation time caused a significant decrease in VCL, VSL and VAP after 30 min (10 mM GSH) and 60 min (4 mM GSH) of incubation, and in LIN after 120 min of incubation (10 mM GSH). Likewise, the incubation time reduced WOB, ALH and BCF at GSH concentrations equal to or higher than 6 mM (*p* ≤ 0.05).

#### 3.1.2. Sperm Viability

**Figure 2:**
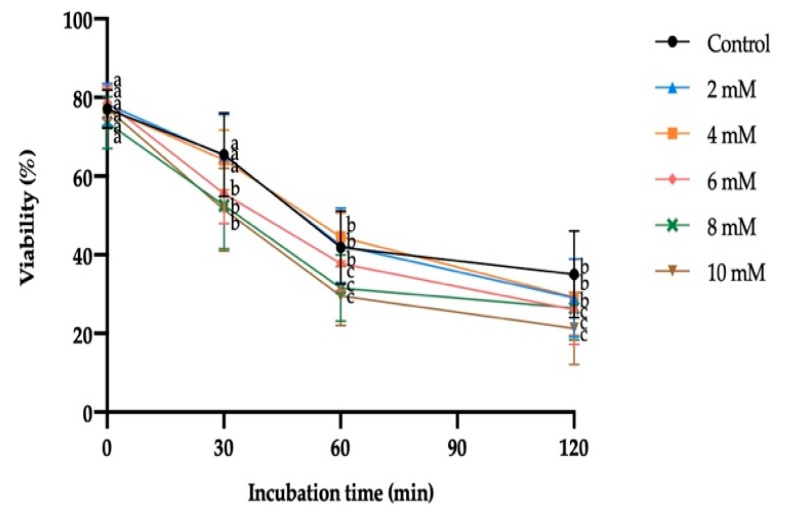
Mean ± SEM of viability of donkey sperm exposed to reductive stress with different reduced glutathione (GSH) concentrations measured at different incubation times. No significant differences were found between GSH concentrations within a given incubation time. (a–c) Different letters indicate significant differences (*p* ≤ 0.05) between incubation times within a given GSH concentration.

Sperm viability was not significantly affected by exposure to GSH at any incubation time or concentration. On the contrary, the incubation time within each treatment produced a significant decrease from 30 min in 6 mM, 8 mM and 10 mM GSH; and from 60 min in the control, 2 mM and 4 mM GSH (Figure 2).

#### 3.1.3. Extracellular Hydrogen Peroxide (H_2_O_2_) Production

**Figure 3:**
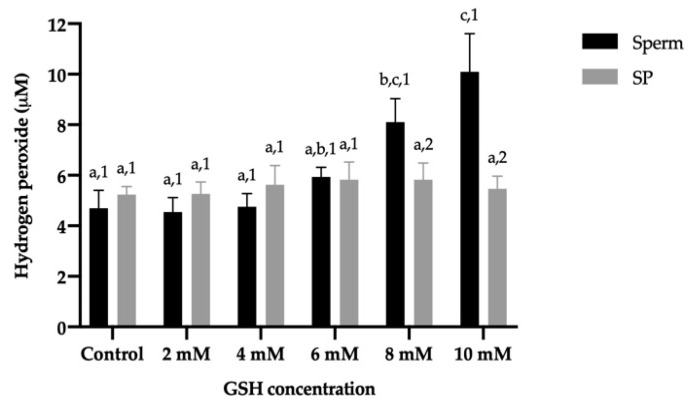
Mean ± SEM of extracellular hydrogen peroxide (H_2_O_2_) produced by donkey sperm and seminal plasma (SP) exposed to reductive stress with increasing reduced glutathione (GSH) concentrations at 37 °C for 30 min. (a–c) Different letters indicate significant differences (*p* ≤ 0.05) between GSH concentrations in sperm or SP. (1,2) Different numbers indicate significant differences (*p* ≤ 0.05) between sperm or SP within a given GSH concentration.

The extracellular H_2_O_2_ production by sperm exposed to reductive stress increased significantly at GSH concentrations equal to or higher than 8 mM. Moreover, the extracellular H_2_O_2_ production by SP was not significantly affected by the different GSH concentrations. Significant differences in the extracellular H_2_O_2_ produced by sperm vs. that produced by SP were only observed in the treatments containing 8 mM or 10 mM GSH (8 mM GSH: 8.11 µM ± 0.93 µM vs. 5.83 µM ± 0.66 µM; 10 mM GSH: 10.09 µM ± 1.51 µM vs. 5.47 µM ± 0.50 µM; Figure 3).

### 3.2. Experiment 1: Exposure of Sperm to Oxidative Stress

#### 3.2.1. Sperm Motility

**Figure 4:**
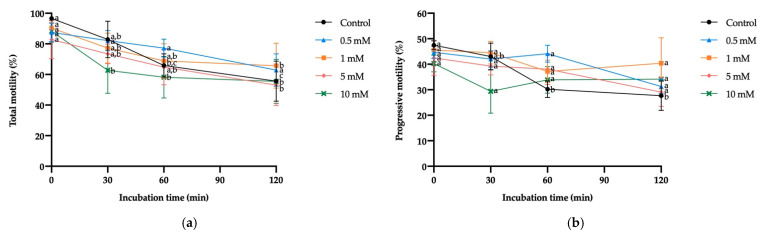
Mean ± SEM of total (**a**) and progressive (**b**) motility of donkey sperm exposed to oxidative stress with different hydrogen peroxide (H_2_O_2_) concentrations measured at different incubation times. No significant differences were found between H_2_O_2_ concentrations within a given incubation time. (**a**,**b**) Different letters indicate significant differences (*p* ≤ 0.05) between incubation times within a given H_2_O_2_ concentration.

No significant differences in TM and PM were found when sperm were exposed to different H_2_O_2_ concentrations, at any of the incubation times. While incubating sperm with 0.5 mM H_2_O_2_ did not affect TM, higher concentrations of H_2_O_2_ decreased that sperm variable (from 30 min in the treatment containing 10 mM H_2_O_2_, and from 120 min in the treatments containing 1 mM and 5 mM H_2_O_2_). Although the incubation time led to a decrease in PM in all treatments, this reduction was only statistically significant (*p* ≤ 0.05) in the control from 60 min of incubation (Figure 4a,b).

Regarding kinematic parameters (Table 2), the presence of 10 mM H_2_O_2_ led to a reduction in sperm velocity (VCL, VSL and VAP) after 30 min of incubation (*p* ≤ 0.05). While LIN, WOB and BCF were not significantly affected by treatment at any of the incubation times, STR increased in 10 mM H_2_O_2_ after 60 min and 120 min of incubation (*p* ≤ 0.05), respectively, and ALH significantly decreased in the treatment containing 5 mM H_2_O_2_ after 30 min of incubation.

The incubation time significantly decreased sperm velocity (VCL and VAP) and ALH; the higher the concentration of H_2_O_2_, the larger the extent of that reduction (from 30 min in 5 mM and 10 mM H_2_O_2_, and from 60 min in 0.5 mM and 1 mM H_2_O_2_). While LIN and STR increased along the incubation time in the treatments containing 5 mM and 10 mM H_2_O_2_ (*p* ≤ 0.05), WOB was not affected. Finally, BCF significantly decreased after 30 min of incubation in the treatment containing 10 mM H_2_O_2_.

#### 3.2.2. Sperm Viability

**Figure 5:**
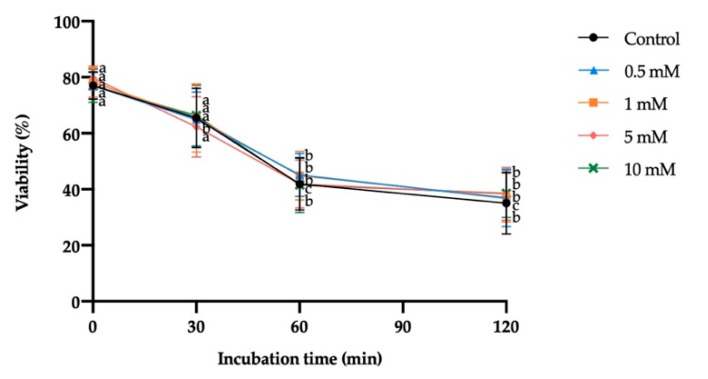
Mean ± SEM of viability of donkey sperm exposed to oxidative stress with different hydrogen peroxide (H_2_O_2_) concentrations measured at different incubation times. No significant differences were found between H_2_O_2_ concentrations within a given incubation time. (a,b) Different letters indicate significant differences (*p* ≤ 0.05) between incubation times within a given H_2_O_2_ concentration.

Exposure to different H_2_O_2_ concentrations did not significantly affect sperm viability at any of the incubation times. However, the incubation time caused a significant decrease in sperm viability regardless of H_2_O_2_ concentration (from 60 min in the control, 0.5 mM, 1 mM and 10 mM H_2_O_2_, and from 30 min in 5 mM H_2_O_2_; Figure 5).

#### 3.2.3. Extracellular Hydrogen Peroxide (H_2_O_2_) Production

No significant differences in the extracellular H_2_O_2_ production were found between the control and treatments in which oxidative stress was induced with H_2_O_2_, either in sperm or in SP. In the same way, the extracellular H_2_O_2_ production in a given treatment did not significantly differ between sperm and SP.

### 3.3. Experiment 2. Extracellular H_2_O_2_ Production by NETosis

#### 3.3.1. Reacted Polymorphonuclear Neutrophils (PMN)

**Figure 6:**
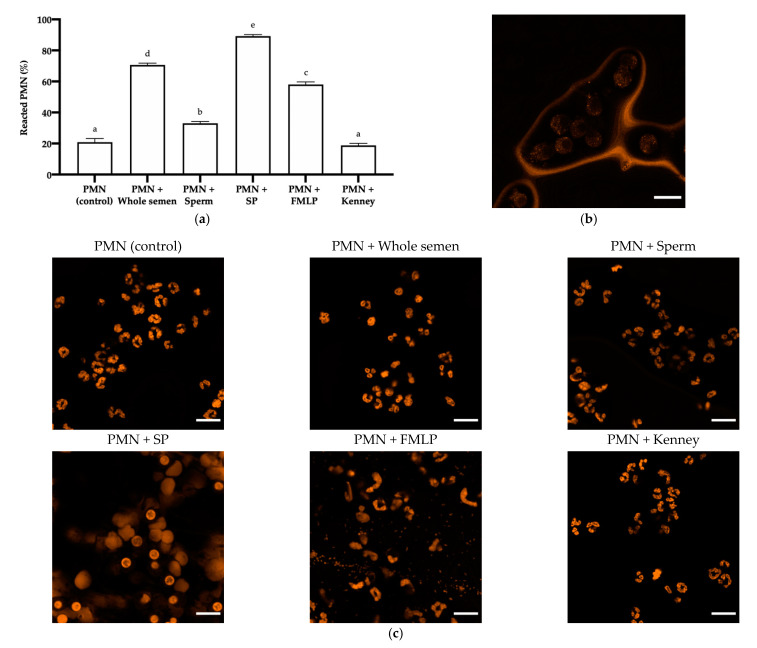
Activation of PMN and NETosis. (**a**) Mean ± SEM of percentages of reacted PMN in each treatment. (a–e) Different letters indicate significant differences (*p* ≤ 0.05) between treatments. (**b**) NETosis of PMN with degranulation/extrusion of their molecular components. (**c**) Representative images resulting from the PMN exposure to the different treatments. Scale bar = 15 µm.

There was a significant effect of the treatment on the percentage of reacted PMN (NETosis; *p* ≤ 0.001; Figure 6a–c). The highest percentage of reacted PMN was found when 25% of SP was added (PMN + SP; 89.19% ± 1.02%), being significantly different from the other treatments. Removing SP from sperm (PMN + Sperm) induced less NETosis than semen containing SP (PMN + Whole semen; 33.03% ± 1.19% vs. 70.65% ± 1.17%; *p* ≤ 0.001). Activation of PMN and, therefore, induction of NETosis was greater in the presence of FMLP (PMN + FMLP) than in both the control (58.07% ± 1.67% vs. 20.91% ± 2.27%; *p* ≤ 0.001) and the treatment containing sperm but not SP (PMN + Sperm; 58.07% ± 1.67% vs. 33.03% ± 1.19%; *p* ≤ 0.001). By contrast, NETosis in the treatments containing SP, either alone (PMN + SP; 89.19% ± 1.02%) or together with sperm (PMN + Sperm; 70.65% ± 1.17%), was significantly higher than in the treatment with FMLP (PMN + FMLP; 58.07% ± 1.67%; *p* ≤ 0.001). Finally, the treatment that included the Kenney extender (PMN + Kenney) did not differ significantly from the control (18.73% ± 1.29% vs. 20.91% ± 2.27%).

#### 3.3.2. Extracellular Hydrogen Peroxide (H_2_O_2_) Production

**Figure 7:**
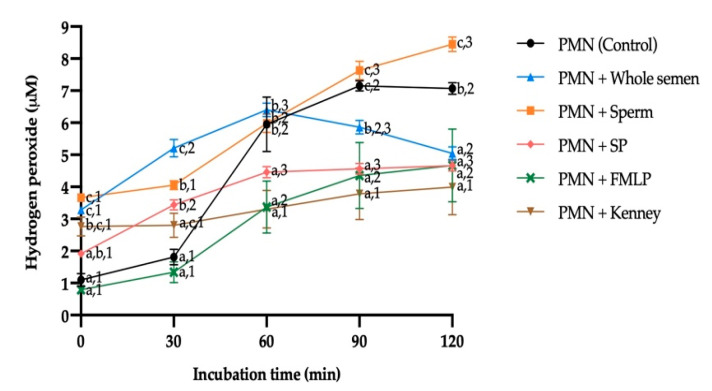
Mean ± SEM of extracellular H_2_O_2_ production of jenny polymorphonuclear neutrophils (PMN) exposed to different treatments. (a–c) Different letters indicate significant differences (*p* ≤ 0.05) between treatments within a given incubation time. (1–3) Different numbers indicate significant differences (*p* ≤ 0.05) between incubation times within a given treatment.

Treatments containing the whole semen (i.e., sperm and SP) generated a significantly higher amount of H_2_O_2_ after 0 min and 30 min of incubation compared with PMN (control), PMN + SP or PMN + FMLP (Figure 7). However, after 30 min of incubation, the treatment containing PMN + SP was found to produce more H_2_O_2_ than the control and the treatment with PMN + FMLP (*p* ≤ 0.05). After 60 min of incubation, PMN (control), PMN + Whole semen and PMN + Sperm presented significantly higher H_2_O_2_ production than the other treatments. Only at 90 min of incubation did the whole semen (PMN + Whole semen) generate less H_2_O_2_ than the control, whereas PMN + Sperm produced more H_2_O_2_ than the control (PMN) after 120 min of incubation (*p* ≤ 0.05).

Regarding the incubation time, the presence of the whole semen and SP significantly increased H_2_O_2_ levels after 30 min of incubation. After 60 min, the control, PMN + Sperm and PMN + FMLP treatments showed significantly higher production of H_2_O_2_ than at the beginning of the experiment (*p* ≤ 0.05). By contrast, production of H_2_O_2_ did not differ along incubation in the treatment containing the Kenney extender (PMN + Kenney).

## 4. Discussion

Intracellular ROS production by sperm mainly results from electron loss in the mitochondrial transport chain during energy generation in the form of ATP [35]. This effect has been associated with greater mitochondrial activity in horse spermatozoa, where the electron loss favors an increase in ROS production even to toxic levels and alters the redox balance [36,37]. Within this context, it is important to mention that the chemical energy required for sperm motility, in the case of the horse, is supplied by the production of mitochondrial ATP derived from OXPHOS [17,18]. As donkey sperm present a significantly higher speed and progressivity compared with horse sperm [38], due to the fact that the former present a larger intermediate piece and a higher mitochondrial membrane potential [39,40] than the latter [41], one could suggest that ROS production by donkey sperm is even higher. However, our results indicate that exposure of donkey sperm to high doses (up to 10 mM) of GSH (reductive stress) and H_2_O_2_ (oxidative stress) does not affect TM, their values being maintained after 60 min and 120 min of incubation. In fact, only a significant reduction in PM was observed at 10 mM GSH. In human sperm, Panner Selvam et al. [23] observed a significant decrease in TM and PM under conditions of reductive and oxidative stress from 30 min of incubation. This would indicate that donkey sperm have great resilience to the toxicity produced by high GSH levels and that their antioxidant capacity is very high. This capacity, together with SP, allows maintaining the redox balance following exposure to high levels of ROS.

When sperm are deposited in the female genital tract, they quickly lose the antioxidant support of SP. From then onward, sperm rely on their own antioxidant defense mechanisms [42], which, in the case of donkeys, are not enough to counteract the powerful oxidative environment of the endometrium due the infiltration of PMN as early as 6–10 h post-AI [3,43]. This leads to the activation of pathways that ultimately result in sperm death (apoptotic-like changes and necrosis after severe stress) [44]. Our results show that as the time of incubation increases, sperm viability is significantly reduced as early as 30 min after the contact occurs. This differs from what is found in humans; in effect, whereas human sperm reduce their viability significantly following exposure to oxidative conditions [23], this is not observed in donkeys because of the resistance of their sperm to high H_2_O_2_ concentrations.

The mechanisms through which donkey sperm are able to tolerate ROS better than antioxidants are still unknown. Surprisingly, we observed that high GSH concentrations caused higher H_2_O_2_ production, contrary to what might be expected once the antioxidant defense system was exogenously enhanced. The link found here could be related to two aspects. The first one would have to do with the presence of the enzyme L-amino acid oxidase (LAAO), which is found in the acrosome of sperm in several species, including humans [45], and is particularly active in the horse [46]. LAAO is responsible for the generation of H_2_O_2_ through deamination of L-amino or aromatic amino acids, such as phenylalanine, tyrosine and tryptophan [47]. In this circumstance, one could reasonably suggest that there could be a relationship between GSH and LAAO, which would support the suggestion that more ROS are produced under reductive conditions. The second aspect that could be envisaged to explain the greater ROS production in the presence of GSH would be the increase in the number of non-viable spermatozoa and even in that of morphologically abnormal ones, which results from incubation. These sperm cells have been shown to contain high levels of H_2_O_2_ synthesized in the mitochondria or in the cytoplasm of the sperm tail, so that they may be a potential source of ROS [48,49]. Furthermore, as non-viable sperm appear to be more sensitive to the presence of L-amino acids than viable cells, ROS production would likely be increased in the presence of non-viable sperm. Taking this aspect into account, we were able to quantify the amount of H_2_O_2_ produced by the non-viable donkey sperm released into the extracellular space, which ranged between 3.94 and 5.20 µM (average = 4.71 µM). The higher extracellular production of H_2_O_2_ began to occur when sperm were exposed to high antioxidant levels (i.e., 8 mM and 10 mM GSH). This makes the initially induced reductive environment become a spontaneous access route for lipid peroxidation in sperm that remain viable due to increased exposure to ROS [49]. Related to this, it has been described that TM and PM decrease significantly in horse sperm as the amount of ROS-producing non-viable sperm increases [50]. However, our CASA observations showed a reduction in sperm motility due to concentrations of GSH ≥ 8 mM after 60 min of incubation, but without this being associated with the presence of non-viable sperm. Furthermore, our findings also suggest that donkey sperm are more resilient to high concentrations of GSH than their horse counterpart, for which 2.5 mM GSH is already harmful [51,52].

The remarkable tolerance to oxidative stress found in our study is possibly an indication of the reproductive strategy developed by the donkey. Contrary to what was thought about the sperm susceptibility to oxidative stress, given by their inadequate cell repair systems and their low capacity for antioxidant defense due to their low cytoplasmic content [53], our data point out to a better antioxidant system in the donkey compared with other species. While the role of the antioxidant enzymes present in donkey SP has been widely described and their activities have been found to be higher than in the horse [26,54], non-enzymatic sperm antioxidants, such as GSH or ascorbic acid (AA), could increase their biological effects in response to the stimulus given by oxidative stress, as previously reported in humans [55]. Paradoxically, we could define the behavior of donkey sperm found in our study as a “type of sperm survival” to oxidative stress. One could speculate that this tolerance would be similar to that observed in cancer cells, which have the ability to detoxify ROS by reconfiguring their metabolic activity, thus favoring their survival in an oxidizing environment and avoiding the activation of known cell death pathways [56]. This generates a broad research field in this animal species that could even serve to take it as a model to better understand what happens in other species. Likewise, the presence of several metabolites that could have a role in the maintenance of ROS balance has recently been described [57] and could become a potential research field in the donkey.

One of the hypotheses put forward in this study was that NETosis is capable of producing much more extracellular ROS (measured in H_2_O_2_ levels) than sperm themselves. While the amount of intracellular H_2_O_2_ produced by donkey sperm can be evaluated through the use of a fluorescent probe sensitive to oxidation (2′,7′-dichlorodihydrofluorescein diacetate; H_2_DCFDA) [39,40], the amount of ROS produced specifically by NETosis is yet to be determined. In this study, NETosis in donkeys was seen to be induced by the presence of SP (either alone or in whole semen), in a similar fashion to that recently reported by Mateo-Otero et al. [30]. In addition, the current work also found that, in the donkey, SP controls extracellular ROS generation and maintains redox balance, as the extracellular H_2_O_2_ production is greater when SP is absent, thus complementing what was recently found by Papas et al. [58], who also demonstrated that the presence of SP improves the relative levels of intracellular superoxides in donkey sperm exposed to high H_2_O_2_ concentrations. This may be due to the fact that even without the existence of a pathogen, PMN are capable of activating one of their spontaneous apoptosis pathways (the intrinsic pathway) in response to a stimulus given by the presence of ROS [59]. This could explain the extracellular H_2_O_2_ levels generated when PMN come into contact with sperm and in the absence of SP, which contains antioxidants. In this scenario, the ROS generated by sperm as a by-product of their metabolism would trigger the intrinsic apoptosis pathway of PMN. In addition to the aforementioned, while PMN were observed to produce little extracellular H_2_O_2_ at the beginning of the experiment, they demonstrated to be capable of generating 5–6 times more extracellular H_2_O_2_ than their initial value along incubation. This effect could be attributed to a spontaneous or constitutive apoptosis process that PMN undergo as a mechanism to maintain the homeostasis of the immune system [60]. Interestingly, the Kenney extender seemed to prevent PMN from reaction, keeping the production of extracellular H_2_O_2_ constant even after 2 h of incubation. In this regard, we posit that one of the components of this extender, glucose, may account for preventing the activation of PMN and thus the release of extracellular ROS. In this sense, the data obtained in humans by Manosudprasit et al. [61] indicate that high concentrations of glucose lead to a significant delay in triggering the spontaneous apoptosis of PMN.

It is worth noting that while exposure to a chemotactic peptide (FMLP) was found to induce a quick activation of donkey PMN, the extent of that induction was larger than that of sperm but lesser than that of SP. Although the role of FMLP as an activating agent for PMN was previously reported by Baumber et al. [21] in horses and by Miró et al. [10] in donkeys, the current study was the first to combine PMN and FMLP without any other cellular component (e.g., sperm). In this scenario, the extracellular H_2_O_2_ production seems to be slower and with levels similar to those produced in the presence of SP. Thus, it could be established that FMLP has an effect similar to that of SP, but only with respect to its interaction with PMN. The comparison of the results obtained in our treatment of PMN + Sperm with those observed in horses [21] indicate that extracellular H_2_O_2_ production was four times higher in the donkey than in the horse after 30 min of incubation. Here, one must point out that the extracellular ROS amount produced by sperm:PMN binding relies upon the PMN concentration [21]. It seems that more PMN lead to more NETosis, resulting in higher extracellular ROS production.

According to the results obtained, it should be considered that PMN could undergo spontaneous apoptosis after 60 min of incubation, with the consequent production of high amounts of extracellular ROS. This would suggest that the activation of any of the cell death pathways, both in sperm and PMN, induced by an oxidative environment, would largely explain these high extracellular H_2_O_2_ levels (4.69 µM ± 0.71 µM in sperm and 5.95 µM ± 0.85 µM in PMN). However, when sperm and PMN were put together, extracellular H_2_O_2_ levels did not increase after 60 min of incubation (PMN + Sperm; 5.98 µM ± 0.29 µM), which would rule out a cumulative effect on extracellular H_2_O_2_ production. Hence, the key factor would appear to be the presence of SP, which could be largely responsible for maintaining the redox balance in any environment (reductive or oxidative) and even in NETosis, keeping extracellular H_2_O_2_ levels almost constant at the same incubation time (60 min; 5.60 µM ± 0.26 µM, 4.81 µM ± 0.24 µM and 4.46 µM ± 0.18 µM, respectively). Furthermore, it was also clear that the absence of SP led to higher amounts of extracellular H_2_O_2_ along the incubation time.

Be that as it may, NETosis is capable of producing an immense amount of ROS, which, in the long run, and if occurs repeatedly and/or excessively, can presumably contribute to chronic inflammation of the donkey endometrium and even end in fibrosis [62]. The action mechanism through which ROS production occurs by NETosis still raises several questions. However, it is known that H_2_O_2_ is produced by a redox reaction (dismutation) of the superoxide anion (O_2_^−^) mediated by the NADPH oxidase (NOX2) complex, generated by the NETs formation, which causes an electron loss of molecular oxygen (O_2_). Finally, this H_2_O_2_ can be converted into hydroxyl anion (OH^−^) through the Harber–Weiss or Fenton reaction [49,63,64,65]. Additionally, for NETosis of PMN to occur as a defense mechanism against pathogens, ROS must be generated through NOX2 [65,66,67]. This substrate is likely provided by both sperm themselves and the uterine environment. However, when NETs are formed, PMN have the ability to capture sperm with the clear objective of eliminating them by NETosis [13,27], possibly this programmed cell death process being the trigger for ROS generation in the extracellular space. Taking into account the fact that NETs formation in donkeys [10] and NETosis itself [30] are dependent on sperm concentration, some spermatozoa could have the ability to avoid their capture in the PMN vicinity, possibly because NETs would already be saturated with spermatozoa, or due to the action of the DNase present in the SP, which would block NETosis [68]. This would, in turn, give a greater number of motile and viable sperm the chance to reach the oviduct and fertilize the oocyte.

## 5. Conclusions

In conclusion, this study shows: (1) that donkey sperm have greater tolerance to oxidative than to reductive stress, as extracellular H_2_O_2_ production is larger at high GSH concentrations; and (2) that while SP is the inducer of NETosis of PMN, it also plays a fundamental role in the redox balance through its antioxidant contribution (enzymatic and non-enzymatic), as it controls the extracellular H_2_O_2_ production from both sperm and PMN. According to the results obtained, and from a practical point of view, the addition of antioxidants such as GSH could be necessary to improve the poor AI outcomes obtained in jennies when using frozen-thawed donkey sperm.

## Figures and Tables

**Table 1:** Mean ± SEM of the kinematic parameters of donkey sperm exposed to reductive stress with different reduced glutathione (GSH) concentrations measured at different incubation times.

Parameter	Incubation Time	GSH Concentration					
		Control	2 mM	4 mM	6 mM	8 mM	10 mM
VCL (µm/s)	0 min	217.05 ± 15.87 ^a,1^	215.20 ± 15.02 ^a,1^	216.39 ± 21.43 ^a,1^	215.58 ± 14.79 ^a,1^	213.17 ± 21.01 ^a,1^	238.79 ± 14.66 ^a,1^
	30 min	200.15 ± 22.28 ^a,1,2^	195.60 ± 11.89 ^a,1^	206.31 ± 15.46 ^a,1^	196.55 ± 22.39 ^a,1^	182.97 ± 25.66 ^a,1,2^	170.67 ± 24.31 ^a,2^
	60 min	156.64 ± 12.80 ^a,2^	180.38 ± 20.14 ^a,1^	170.12 ± 21.63 ^a,1^	183.45 ± 16.87 ^a,1^	181.28 ± 21.96 ^a,1,2^	195.84 ± 24.81 ^a,1,2^
	120 min	155.60 ± 18.77 ^a,2^	186.70 ± 14.20 ^a,1^	173.22 ± 9.72 ^a,1^	189.96 ± 28.20 ^a,1^	156.98 ± 13.22 ^a,2^	156.29 ± 15.83 ^a,2^
VSL (µm/s)	0 min	80.68 ± 2.37 ^a,1^	82.30 ± 5.91 ^a,1^	84.67 ± 5.17 ^a,1^	83.38 ± 2.61 ^a,1^	80.77 ± 5.80 ^a,1^	88.24 ± 5.33 ^a,1^
	30 min	78.37 ± 4.47 ^a,1^	78.99 ± 2.04 ^a,1^	82.48 ± 1.65 ^a,1,2^	71.68 ± 8.34 ^a,1^	58.79 ± 7.20 ^a,1,2^	63.45 ± 8.09 ^a,2^
	60 min	60.96 ± 5.56 ^a,1^	68.60 ± 6.05 ^a,1^	60.90 ± 9.28 ^a,2^	64.44 ± 5.76 ^a,1^	58.77 ± 7.45 ^a,1,2^	68.01 ± 8.01 ^a,1,2^
	120 min	65.40 ± 7.04 ^a,1^	71.73 ± 3.22 ^a,1^	63.63 ± 7.24 ^a,1,2^	66.79 ± 15.92 ^a,1^	51.90 ± 6.35 ^a,2^	48.37 ± 5.43 ^a,2^
VAP (µm/s)	0 min	113.58 ± 6.16 ^a,1^	112.78 ± 9.07 ^a,1^	114.13 ± 10.00 ^a,1^	111.58 ± 5.76 ^a,1^	107.90 ± 9.10 ^a,1^	120.83 ± 6.54 ^a,1^
	30 min	107.14 ± 9.62 ^a,1,2^	105.92 ± 2.58 ^a,1^	106.83 ± 4.72 ^a,1,2^	96.37 ± 12.06 ^a,1^	84.47 ± 12.31 ^a,1,2^	83.08 ± 10.96 ^a,2^
	60 min	81.07 ± 5.28 ^a,2^	92.39 ± 9.73 ^a,1^	82.99 ± 12.00 ^a,2^	85.29 ± 8.37 ^a,1^	85.48 ± 9.67 ^a,1,2^	90.76 ± 11.20 ^a,1,2^
	120 min	83.48 ± 8.21 ^a,2^	89.85 ± 5.75 ^a,1^	83.80 ± 6.50 ^a,2^	87.68 ± 15.96 ^a,1^	72.10 ± 6.39 ^a,2^	68.19 ± 8.61 ^a,2^
LIN (%)	0 min	34.97 ± 1.52 ^a,1^	35.28 ± 1.46 ^a,1^	36.36 ± 0.60 ^a,1^	35.71 ± 1.04 ^a,1^	34.52 ± 0.80 ^a,1^	34.72 ± 0.26 ^a,1^
	30 min	36.48 ± 1.57 ^a,1^	37.25 ± 2.66 ^a,1^	36.68 ± 1.88 ^a,1^	34.13 ± 1.44 ^ab,1^	28.57 ± 0.99 ^b,1^	33.57 ± 3.44 ^ab,1,2^
	60 min	35.03 ± 2.70 ^a,1^	35.10 ± 1.06 ^a,1^	32.21 ± 1.67 ^a,1^	31.64 ± 1.30 ^a,1^	29.24 ± 1.48 ^a,1^	32.15 ± 0.67 ^a,1,2^
	120 min	37.76 ± 2.92 ^a,1^	34.68 ± 1.02 ^ab,1^	33.56 ± 2.16 ^ab,1^	31.19 ± 4.82 ^ab,1^	29.02 ± 2.69 ^b,1^	27.64 ± 1.47 ^b,2^
STR (%)	0 min	65.16 ± 1.31 ^a,1^	66.17 ± 0.87 ^a,1^	67.88 ± 0.54 ^a,1^	67.87 ± 0.56 ^a,1^	66.95 ± 1.24 ^a,1^	67.31 ± 0.83 ^a,1^
	30 min	66.55 ± 1.30 ^a,1^	67.52 ± 2.44 ^a,1^	69.53 ± 1.69 ^a,1^	67.52 ± 2.35 ^a,1^	61.26 ± 0.82 ^a,1^	67.23 ± 3.30 ^a,1^
	60 min	65.18 ± 3.11 ^a,1^	66.64 ± 0.44 ^a,1^	64.29 ± 1.93 ^a,1^	66.75 ± 1.32 ^a,1^	61.41 ± 3.81 ^a,1^	67.98 ± 1.41 ^a,1^
	120 min	68.78 ± 3.23 ^a,1^	70.73 ± 1.80 ^a,1^	67.47 ± 4.22 ^a,1^	65.57 ± 8.39 ^a,1^	61.69 ± 3.97 ^a,1^	63.32 ± 4.37 ^a,1^
WOB (%)	0 min	51.44 ± 1.43 ^a,1^	51.33 ± 1.71 ^a,1,2^	51.45 ± 1.17 ^a,1^	50.60 ± 1.49 ^a,1^	49.33 ± 0.68 ^a,1^	49.78 ± 0.38 ^a,1^
	30 min	52.36 ± 1.38 ^a,1^	52.79 ± 1.96 ^a,1^	50.50 ± 1.43 ^a,1^	48.62 ± 1.67 ^ab,1,2^	44.94 ± 1.30 ^b,1^	47.75 ± 2.43 ^ab,1,2^
	60 min	50.78 ± 1.74 ^a,1^	50.31 ± 0.97 ^a,1,2^	47.92 ± 1.08 ^a,1^	46.07 ± 0.83 ^a,1,2^	47.40 ± 1.09 ^a,1^	46.98 ± 0.84 ^a,1,2^
	120 min	52.23 ± 1.88 ^a,1^	47.48 ± 2.23 ^ab,2^	48.76 ± 0.59 ^ab,1^	45.70 ± 1.78 ^b,2^	47.24 ± 0.81 ^ab,1^	43.59 ± 1.96 ^b,2^
ALH (µm)	0 min	2.56 ± 0.14 ^a,1^	2.58 ± 0.16 ^a,1^	2.63 ± 0.22 ^a,1^	2.73 ± 0.19 ^a,1^	2.77 ± 0.24 ^a,1^	2.96 ± 0.15 ^a,1^
	30 min	2.34 ± 0.24 ^a,1,2^	2.40 ± 0.17 ^a,1^	2.63 ± 0.17 ^a,1^	2.59 ± 0.24 ^a,1^	2.44 ± 0.26 ^a,1,2^	2.25 ± 0.29 ^a,1,2^
	60 min	2.00 ± 0.16 ^a,1,2^	2.29 ± 0.20 ^a,1^	2.22 ± 0.21 ^a,1^	2.45 ± 0.17 ^a,1^	2.40 ± 0.24 ^a,1,2^	2.58 ± 0.29 ^a,1,2^
	120 min	1.93 ± 0.22 ^a,2^	2.45 ± 0.17 ^a,1^	2.24 ± 0.07 ^a,1^	2.51 ± 0.32 ^a,1^	2.12 ± 0.15 ^a,2^	2.20 ± 0.20 ^a,2^
BCF (Hz)	0 min	34.39 ± 1.42 ^a,1^	33.21 ± 3.26 ^a,1^	33.27 ± 2.16 ^a,1^	31.41 ± 2.26 ^a,1^	28.45 ± 1.49 ^a,1^	32.58 ± 1.06 ^a,1^
	30 min	35.00 ± 2.38 ^a,1^	34.77 ± 1.55 ^a,1^	31.43 ± 0.60 ^ab,1^	28.21 ± 3.29 ^abc,1,2^	22.44 ± 2.19 ^c,1,2^	24.09 ± 2.25 ^bc,2^
	60 min	28.13 ± 1.49 ^ab,1^	29.75 ± 2.70 ^a,1^	26.33 ± 3.35 ^ab,1^	22.67 ± 2.22 ^ab,2^	20.62 ± 2.14 ^b,2^	21.67 ± 1.69 ^ab,2^
	120 min	31.29 ± 1.86 ^a,1^	28.78 ± 2.04 ^ab,1^	27.64 ± 1.60 ^abc,1^	27.12 ± 2.19 ^abc,1,2^	23.04 ± 1.28 ^bc,1,2^	20.55 ± 1.99 ^c,2^

VCL (µm/s): curvilinear velocity; VSL (µm/s): straight line velocity; VAP (µm/s): average path velocity; LIN (%): linearity coefficient; STR (%): straightness coefficient; WOB (%): wobble coefficient; ALH (µm): amplitude of lateral head displacement; BCF (Hz): beat-cross frequency. (^a–c^) Different letters indicate significant differences (*p* ≤ 0.05) between GSH concentrations within a given incubation time. (^1,2^) Different numbers indicate significant differences (*p* ≤ 0.05) between incubation times within a given GSH concentration.

**Table 2:** Mean ± SEM of the kinematic parameters of donkey sperm exposed to oxidative stress with different hydrogen peroxide (H_2_O_2_) concentrations measured at different incubation times.

Parameter	Incubation Time	H_2_O_2_ Concentration				
		Control	0.5 mM	1 mM	5 mM	10 mM
VCL (µm/s)	0 min	217.05 ± 15.87 ^a,1^	207.18 ± 12.71 ^a,1^	198.34 ± 15.79 ^a,1^	200.12 ± 19.14 ^a,1^	200.68 ± 13.72 ^a,1^
	30 min	200.15 ± 22.28 ^a,1^	173.46 ± 15.47 ^ab,1,2^	170.15 ± 4.75 ^ab,1,2^	154.00 ± 10.13 ^b,2^	152.15 ± 11.91 ^b,2^
	60 min	156.64 ± 12.80 ^a,2^	158.57 ± 12.00 ^a,2^	146.71 ± 8.83 ^a,2^	152.04 ± 13.70 ^a,2^	164.64 ± 15.75 ^a,1,2^
	120 min	155.60 ± 18.77 ^a,2^	154.92 ± 15.31 ^a,2^	167.50 ± 12.09 ^a,1,2^	160.25 ± 16.59 ^a,2^	169.35 ± 11.85 ^a,1,2^
VSL (µm/s)	0 min	80.68 ± 2.37 ^a,1^	78.03 ± 2.08 ^a,1^	76.12 ± 3.37 ^a,1^	76.52 ± 5.10 ^a,1^	72.05 ± 2.40 ^a,1,2^
	30 min	78.37 ± 4.47 ^a,1^	69.56 ± 3.33 ^ab,1^	74.73 ± 4.44 ^ab,1^	66.62 ± 4.12 ^ab,1^	56.47 ± 4.02 ^b,2^
	60 min	60.96 ± 5.56 ^a,2^	67.32 ± 3.36 ^a,1^	63.29 ± 2.09 ^a,1^	68.86 ± 7.00 ^a,1^	72.61 ± 6.91 ^a,1,2^
	120 min	65.40 ± 7.04 ^a,1,2^	62.58 ± 6.28 ^a,1^	74.44 ± 7.67 ^a,1^	68.07 ± 8.25 ^a,1^	78.77 ± 5.25 ^a,1^
VAP (µm/s)	0 min	113.58 ± 6.16 ^a,1^	108.23 ± 5.43 ^a,1^	104.02 ± 6.23 ^a,1^	104.42 ± 8.38 ^a,1^	103.43 ± 5.96 ^a,1^
	30 min	107.14 ± 9.62 ^a,1^	92.98 ± 5.55 ^ab,1,2^	94.95 ± 3.21 ^ab,1,2^	86.27 ± 4.41 ^ab,1^	79.06 ± 5.80 ^b,2^
	60 min	81.07 ± 5.28 ^a,2^	86.72 ± 4.68 ^a,1,2^	81.46 ± 3.70 ^a,2^	85.99 ± 8.26 ^a,1^	90.97 ± 8.25 ^a,1,2^
	120 min	83.48 ± 5.28 ^a,2^	81.37 ± 4.68 ^a,2^	92.09 ± 3.70 ^a,1,2^	86.20 ± 8.26 ^a,1^	94.32 ± 8.25 ^a,1,2^
LIN (%)	0 min	34.97 ± 1.52 ^a,1^	34.94 ± 1.24 ^a,1^	35.72 ± 1.65 ^a,1^	34.97 ± 0.82 ^a,2^	33.46 ± 1.65 ^a,2^
	30 min	36.48 ± 1.57 ^a,1^	36.71 ± 1.58 ^a,1^	40.58 ± 2.68 ^a,1^	39.94 ± 2.14 ^a,1,2^	34.51 ± 2.65 ^a,2^
	60 min	35.03 ± 2.70 ^a,1^	40.24 ± 1.24 ^a,1^	40.98 ± 0.84 ^a,1^	42.22 ± 1.99 ^a,1^	42.12 ± 3.12 ^a,1^
	120 min	37.76 ± 2.92 ^a,1^	36.87 ± 3.50 ^a,1^	41.35 ± 3.45 ^a,1^	39.34 ± 4.26 ^a,1,2^	43.57 ± 3.93 ^a,1^
STR (%)	0 min	65.16 ± 1.31 ^a,1^	65.42 ± 1.00 ^a,1^	66.39 ± 1.52 ^a,1^	65.97 ± 0.26 ^a,1^	63.27 ± 1.40 ^a,2^
	30 min	66.55 ± 1.30 ^a,1^	66.80 ± 1.10 ^a,1^	71.07 ± 2.81 ^a,1^	68.81 ± 1.94 ^a,1^	63.96 ± 1.42 ^a,2^
	60 min	65.18 ± 3.11 ^b,1^	70.34 ± 0.97 ^ab,1^	70.19 ± 1.79 ^ab,1^	71.65 ± 1.93 ^ab,1^	73.34 ± 3.38 ^a,1^
	120 min	68.78 ± 3.23 ^ab,1^	67.96 ± 3.64 ^b,1^	73.13 ± 3.40 ^ab,1^	70.40 ± 4.01 ^ab,1^	75.85 ± 3.37 ^a,1^
WOB (%)	0 min	51.44 ± 1.43 ^a,1^	51.23 ± 1.21 ^a,1^	51.58 ± 1.52 ^a,1^	50.96 ± 1.31 ^a,1^	50.53 ± 1.71 ^a,1^
	30 min	52.36 ± 1.38 ^a,1^	52.48 ± 1.60 ^a,1^	54.73 ± 1.68 ^a,1^	55.55 ± 1.91 ^a,1^	51.80 ± 2.69 ^a,1^
	60 min	50.78 ± 1.74 ^a,1^	54.86 ± 1.26 ^a,1^	56.07 ± 0.70 ^a,1^	56.43 ± 1.19 ^a,1^	55.49 ± 2.11 ^a,1^
	120 min	52.23 ± 1.88 ^a,1^	51.69 ± 2.34 ^a,1^	54.27 ± 2.86 ^a,1^	53.03 ± 2.99 ^a,1^	55.20 ± 3.08 ^a,1^
ALH (µm)	0 min	2.56 ± 0.14 ^a,1^	2.51 ± 0.14 ^a,1^	2.44 ± 0.18 ^a,1^	2.45 ± 0.23 ^a,1^	2.43 ± 0.15 ^a,1^
	30 min	2.34 ± 0.24 ^a,1,2^	2.15 ± 0.20 ^ab,1,2^	2.06 ± 0.05 ^ab,1,2^	1.86 ± 0.12 ^b,2^	1.87 ± 0.14 ^b,2^
	60 min	2.00 ± 0.16 ^a,2,3^	1.95 ± 0.14 ^a,2^	1.79 ± 0.09 ^a,2^	1.81 ± 0.12 ^a,2^	1.98 ± 0.16 ^a,2^
	120 min	1.93 ± 0.22 ^a,3^	1.92 ± 0.16 ^a,2^	2.02 ± 0.10 ^a,2^	1.96 ± 0.16 ^a,2^	1.98 ± 0.13 ^a,2^
BCF (Hz)	0 min	34.39 ± 1.42 ^a,1^	32.23 ± 2.03 ^a,1^	32.46 ± 1.94 ^a,1^	31.76 ± 2.47 ^a,1^	32.01 ± 1.72 ^a,1,2^
	30 min	35.00 ± 2.38 ^a,1^	32.29 ± 1.10 ^a,1^	34.61 ± 1.42 ^a,1^	32.33 ± 1.29 ^a,1^	27.94 ± 3.34 ^a,2^
	60 min	28.13 ± 1.49 ^a,1^	32.00 ± 0.81 ^a,1^	32.33 ± 0.81 ^a,1^	34.66 ± 2.09 ^a,1^	34.01 ± 1.68 ^a,1,2^
	120 min	31.29 ± 1.86 ^a,1^	31.27 ± 2.46 ^a,1^	36.27 ± 3.54 ^a,1^	33.28 ± 3.36 ^a,1^	38.17 ± 2.71 ^a,1^

VCL (µm/s): curvilinear velocity; VSL (µm/s): straight line velocity; VAP (µm/s): average path velocity; LIN (%): linearity coefficient; STR (%): straightness coefficient; WOB (%): wobble coefficient; ALH (µm): amplitude of lateral head displacement; BCF (Hz): beat-cross frequency. (^a,b^) Different letters indicate significant differences (*p* ≤ 0.05) between H_2_O_2_ concentrations within a given incubation time. (^1–3^) Different numbers indicate significant differences (*p* ≤ 0.05) between incubation times within a given H_2_O_2_ concentration.

## Data Availability

All data is contained within the article and supplementary materials.

## Supplementary Materials

The following are available online at https://www.mdpi.com/article/10.3390/antiox10091367/s1 (accessed on 17 August 2021).
